# Synthesizing the links between secure housing tenure and health for more equitable cities

**DOI:** 10.12688/wellcomeopenres.17244.2

**Published:** 2023-03-13

**Authors:** Jill Baumgartner, Judith Rodriguez, Frans Berkhout, Yvonne Doyle, Majid Ezzati, George Owusu, Zahidul Quayyum, Bethlehem Solomon, Meghan Winters, Gary Adamkiewicz, Brian E. Robinson

**Affiliations:** 1 Institute for Health and Social Policy, McGill University, Montreal, Canada; 2 Department of Epidemiology, Biostatistics, and Occupational Health, McGill University, Montreal, Canada; 3 Department of Epidemiology and Biostatistics, Imperial College London, London, UK; 4 Graduate School of Design, Harvard University, Cambridge, USA; 5 Department of Environmental Health, Harvard T.H. Chan School of Public Health, Boston, USA; 6 Department of Geography, Faculty of Social Science & Public Policy, King’s College London, London, UK; 7 Public Health England, London, UK; 8 Regional Institute for Population Studies, University of Ghana, Accra, Ghana; 9 Institute of Statistical, Social and Economic Research, University of Ghana, Accra, Ghana; 10 Centre for Urban Management Studies, University of Ghana, Accra, Ghana; 11 James P Grant School of Public Health, BRAC University, Dhaka, Bangladesh; 12 Faculty of Health Sciences, Simon Fraser University, Vancouver, Canada; 13 Department of Geography, McGill University, Montreal, Canada

**Keywords:** environment, housing, neighbourhood, pollution, slums, sustainability, urban health

## Abstract

Millions of households in rich and poor countries alike are at risk of being unwilfully displaced from their homes or the land on which they live (i.e., lack secure tenure), and the urban poor are most vulnerable. Improving housing tenure security may be an intervention to improve housing and environmental conditions and reduce urban health inequalities. Building on stakeholder workshops and a narrative review of the literature, we developed a conceptual model that infers the mechanisms through which more secure housing tenure can improve housing, environmental quality, and health. Empirical studies show that more secure urban housing tenure can boost economic mobility, improve housing and environmental conditions including reduced exposure to pollution, create safer and more resourced communities, and improve physical and mental health. These links are shared across tenure renters and owners and different economic settings. Broader support is needed for context-appropriate policies and actions to improve tenure security as a catalyst for cultivating healthier homes and neighbourhoods and reducing urban health inequalities in cities.

## Introduction

The number of people and the proportion of the world’s population living in cities have rapidly increased, with an estimated 4.4 billion urban residents comprising 56% of the global population ( United Nations, 2018). Urban residents have better average health than their rural counterparts ( Dye, 2008), but these health advantages are often unevenly distributed where large inequalities in environmental and living conditions exist across small spatial scales ( Bennett *et al.,* 2015; Günther & Harttgen, 2012; Henderson *et al.,* 2016). Most city-level health interventions focus on singular risk factors and health outcomes in very specific populations ( Corburn, 2009), but reducing urban health inequalities requires intersectoral actions that address multiple sectors, multiple social and environmental risks, and multiple health outcomes ( Brown *et al.,* 2019).

Housing is one such factor that affects multiple aspects of living and environmental conditions and health. In particular, how secure people feel about their housing and land tenure affects their confidence that they will reap the medium- or longer-term benefits from investment made in property today ( Rolnik, 2012; Tseng *et al.,* 2021), which has implications for the quality of the property and its neighbourhood. Millions of households in rich and poor countries alike do not feel secure in their tenure – that is, they live with the threat of being unwilfully displaced from their homes or the land on which they live ( Prindex, 2019), and equity in property tenure is a target of two Sustainable Development Goals (SDGs) (1.4.2 and 5.a.1). Yet tenure security has historically been overlooked in policy realms as a health intervention (c.f., Decent Housing Program and The Health Foundation in the U.K. ( Parliamentary Office for Science and Technology, 2011; The Health Foundation, 2021) and the World Health Organization (WHO) Housing and Health Guidelines which specifically mention the importance of secure tenure for mental health ( WHO, 2018)).

Cities represent complex systems and, within them, housing and health depend on many interactions where causation is non-linear ( Baker *et al.,* 2017), but this is not well-reflected in decades of topic-based research. One likely reason is that topics of tenure security, housing, environment, and health are often academically and professionally siloed ( Crawford *et al.,* 2010; Lawrence, 2004). Considerable progress has been made to link tenure security and livelihoods ( Tseng *et al.,* 2021) and, separately, housing and environmental risks with health ( Adamkiewicz *et al.,* 2011; Moloughney, 2004; WHO, 2018). Yet most did not explicitly leverage or acknowledge the links that connect tenure security and health. What is needed is a linking of these evidence bases to illustrate how tenure security underpins key housing, social, and environmental risks, and their associated health and wellbeing outcomes.

We identified housing tenure security as a multi-sectoral urban health priority through a co-production of research approach that drew on expert opinions from academic and policy spheres. We then conducted a narrative review of empirical research and held discussions among researchers to inform a conceptual model that infers the mechanisms through which the provision of more secure tenure can improve urban health across a range of tenure forms and geographic settings. Finally, we outlined some policies and actions than can be taken by cities to improve housing tenure security across different forms of tenure and levels of development. 

## What is meant by secure housing tenure?

“Housing tenure” describes the bundle of rights that an occupant has over the property where they live. For example, does one have the right of alienation (i.e., the right to sell or sub-divide)? Does one have the right to bequest? The nature of these rights is often referred to as the *substance* of tenure ( Arnot *et al.,* 2011; Sjaastad & Bromley, 2000). Substance involves the size of the bundle of rights and the duration of those rights, which is how we define categories of different forms of tenure (i.e., occupant as an owner, renter, stayer, or squatter). Importantly, the categories that describe the form of tenure are distinct from the *security* of that tenure, the latter being the focus of this paper.

Security in housing tenure generally refers to the chance that the bundle of rights held by an occupant will be recognized and upheld by society ( Sjaastad & Bromley, 2000). A lack of assurance – that is, to be housing insecure – implies that the occupants perceive some risk in losing some or all rights associated with the place they live.

How secure one feels in their tenure relates various contextual and individual factors. These can be broadly categorized into surrounding political economy (prices, economic pressures, etc.), formal institutions (laws, governance, enforcement, etc.), and informal institutions (perceptions, norms, etc.) ( Robinson *et al.,* 2018). Thus, occupants’ perceptions, cultural contexts, and legal claim can all play key roles in housing investment decisions ( Durand-Lasserve & Royston, 2002; Robinson *et al.,* 2018). For these reasons an exact definition of secure tenure is widely debated, but what is clear is that housing tenure security is a major challenge for cities. Newly released data on housing tenure security in 33 mostly low- and middle-income countries are striking: nearly one in four urban dwellers perceived their housing tenure as insecure ( Prindex, 2019).

## Methods

Our paper and conceptual model were motivated by stakeholder workshops convened in Vancouver, Canada (June, 2017) and Accra, Ghana (May and October, 2019) through the *Pathways to Equitable Healthy Cities* research consortium ( http://equitablehealthycities.org). Attendees included consortium researchers in health, engineering, and social sciences and practitioners from government and civil society that worked in the urban planning, business development, housing, energy, water, transportation, and health sectors. In these workshops, practitioners identified challenges and opportunities for healthier urban development in their cities, but without any framing by researchers. Researchers listened and gained perspectives on policy and economic priorities, social dimensions, and equity issues for cities. Detailed information about the knowledge co-production exercises and outcomes are published elsewhere ( Audia *et al.,* 2021).

Housing and land tenure security emerged as a multi-sectoral policy priority, and one for which researchers and practitioners could benefit from a conceptual model to navigate the many factors and exposures that link tenure security and health. Next, these links were used as topics for a subsequent narrative literature review, and then iteratively adapted based on feedback from *Pathways* consortium members. Broadly, our review aimed to synthesize the established links across diverse settings and support an encompassing and universal framework that describes the links between tenure security and health. We especially looked for reviews and studies with more rigorous causal inference designs including randomized and quasi-randomized approaches but kept our search wide for key descriptive and associational studies. 

To provide a global perspective, we visualized published data on proportion of population with perceived tenure security by tenure form and income level from over 53,000 households in 33 countries in 2018 using a publicly-available dataset from the Property Rights Index (Prindex) initiative ( https://www.prindex.net/data/). Details on the questionnaires and sampling strategy are provided elsewhere ( Prindex, 2019). Questions used to assess perceived tenure security are shown in Appendix Table A.1. Data visualization was performed in R ( http://www.R-project.org).

## Results

### Conceptual model linking housing tenure security and health

**Figure 1.**
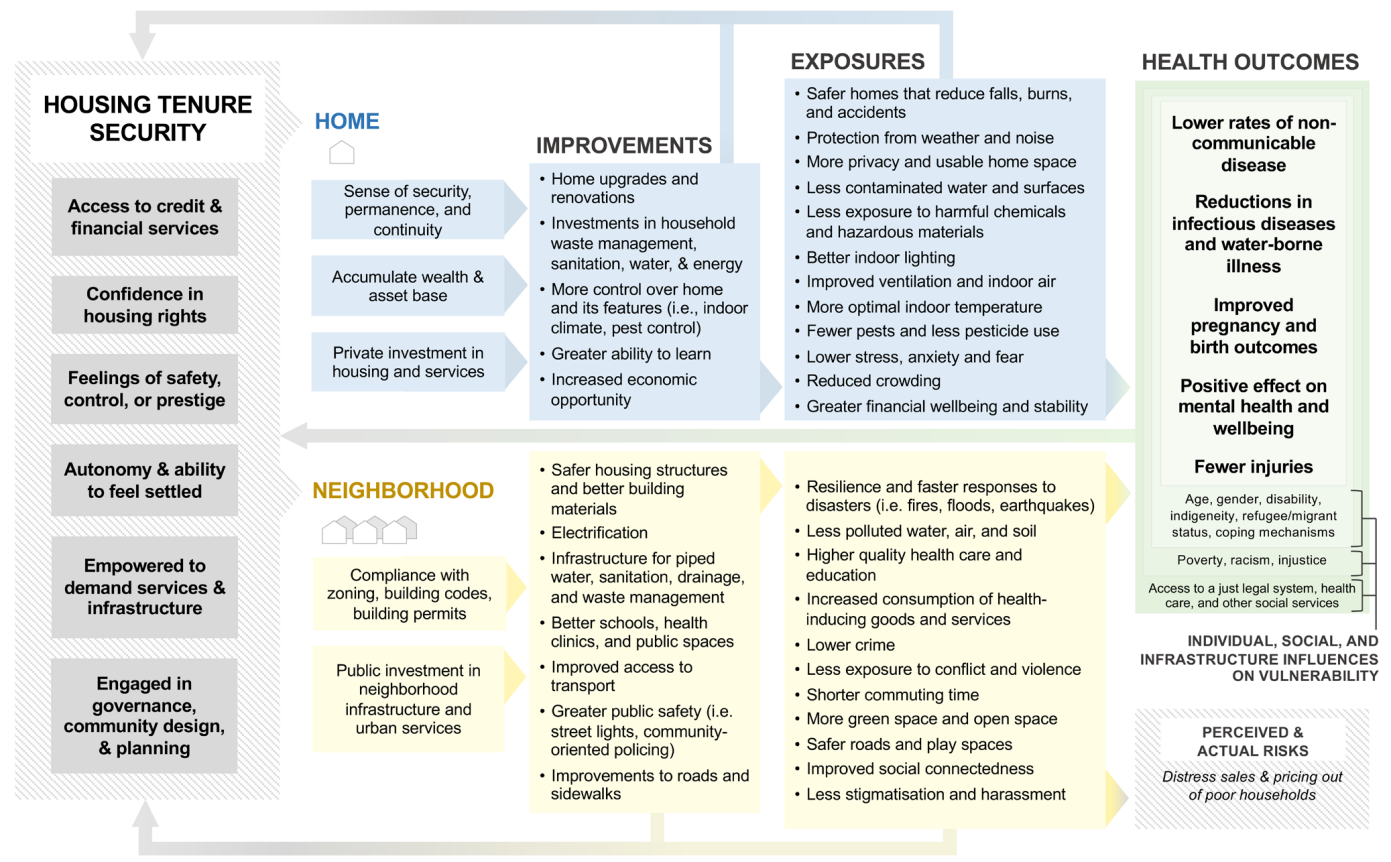
Conceptual model of the linkages between housing tenure security and health ^a^. ^a^A version of the conceptual model with references can be found in Appendix Figure A1.

***Overview of the model.*** Figure 1 presents a conceptual model of a hypothesized chain of events through which housing tenure security can affect home and neighbourhood factors that impact urban health, which is the output of our extensive co-production exercises and comprehensive narrative review. Home factors include both physical features of homes (i.e., structural and design features including space, warmth, dryness, sanitation) and the psychosocial and economic aspects of housing including security, control, sense of attachment, permanence, and continuity. Neighbourhood factors include the location of the home and the provision of urban infrastructure and services including roads, schools, health care, and recreation activities ( Moloughney, 2004).

Our model describes the benefits of households and communities having more secure tenure, which includes greater autonomy, feelings of safety and control, and better access to credit and other financial resources. Tenure security can empower households and communities to demand better infrastructure and urban services and to engage in local governance. These factors can facilitate *improvements* to home and neighbourhood safety and quality of infrastructure and increase access to urban services, which can reduce *exposures* to environmental, social, and housing risks (e.g., flooding, pests, pollution, crime and violence, unsafe roads and play areas) that have well-established impacts on health. The benefits of secure tenure may not be the same for all population groups where individual (e.g., age, gender, disability, race/ethnicity, indigeneity), social (e.g., racism and other oppressive structures, poverty), and infrastructure (e.g., health care) factors can influence vulnerability independently or jointly and thus affect the potential health benefits gained from more secure tenure, as has been demonstrated in studies of ecosystem change and health ( Myers & Patz, 2009). These housing and community upgrades can also have *indirect and dynamic consequences*. For example, improving neighbourhood quality can increase tenure security, but without adequate protections in place, these improvements may lead to perceived or actual gentrification and the pricing-out of poor households in the medium-term, which can reduce tenure security.

To ease the interpretability of our conceptual model, improvements and exposures were separated and categorized, where improvements describe the physical, economic, and social changes and upgrades to homes and neighborhoods and exposures describe the downstream risk factors for health that can be affected by these changes. This simplified model does not capture all interactions, bidirectionalities, or feedbacks between individual improvements or exposures (i.e., privacy could also affect stress and anxiety; reduced crowding could affect ability to learn; opposite to how we have drawn in Figure 1). Rather, we included general feedback loops to indicate that home and neighborhood improvements, reduced social and environmental risks, or improved health can all influence tenure security.

The conceptual model provides generalized insights into the mechanisms linking tenure security and health without being overly prescriptive about the specific policy solutions or interventions, since these are likely to vary across and even within cities and regions. We intend for it to be general enough to apply broadly across contexts while acknowledging that the relative importance of different pathways is likely to differ greatly by place or population. Key pathways to improve health outcomes in, say, a post-industrial city in North America may differ from informal settlements in the Global South. 

***Linking secure tenure with improved livelihoods and living conditions.*** *Low- and middle-income country contexts:* Secure housing and land tenure helps reduce poverty and improve living conditions. Evidence is widespread that secure tenure boosts economic mobility and development and enables households to more confidently invest in and manage their properties for longer-term benefits ( Fenske, 2011; Van Gelder, 2007). Insecure tenure, by contrast, can stem from various sources. For example, constant threats of eviction by the city was a key barrier to investment in environmental infrastructure and repressed livelihoods in informal neighbourhoods of Accra ( Awumbila *et al.,* 2014; Nyametso, 2012). Greater tenure security, however, is associated with improved educational outcomes, access to credit, participation in market activity, and overall wealth in informal settings ( Durand-Lasserve & Royston, 2002; Galiani & Schargrodsky, 2010). In Lima, Peru, acquisition of legal title reduced child labour and increased adult work hours by freeing up time that had been previously used to protect against eviction compared with controls ( Field, 2007).

Historically, ownership through legal title was the focus of most urban tenure policies. Evaluations of titling and registration programs indicate that titled households are more likely to invest in home improvements including piped water connections, waste removal, better heating systems and home ventilation, and soil remediation ( Shaw, 2004). Title can be capitalized as an asset to facilitate, for example, sale of a current house and purchase of a new one to accommodate growing families and avoid overcrowding ( Galiani & Schargrodsky, 2010). Strengthening property rights in slum neighbourhoods in Accra, Lima, and Buenos Aires, Argentina, led to increased household investments in sanitation and electricity and higher rates of home renovation ( Awumbila *et al.,* 2014; Field, 2005; Galiani & Schargrodsky, 2010).

Beyond formalized ownership and land title, studies indicate that households that simply *feel* more tenure secure are more likely to contract environmental services (e.g., waste removal, water, sanitation) and pursue infrastructure upgrades and self-built construction, independent of ownership ( Payne, 2005; Scott *et al.,* 2013; Van Gelder, 2013).

Still, property title and homeownership clearly relate to more secure tenure, even if they are not sufficient for security. In Accra, utility companies require proof of homeownership before connecting households to electricity and water services ( Awumbila *et al.,* 2014). Residents of informal communities in Beijing (China) reported a high degree of perceived tenure security but lack of registered title decreased willingness to invest in home and neighbourhood improvements, indicating that legal title matters more for investment in the property than perceived tenure security in some settings ( Wang *et al.,* 2014).

**Figure 2.**
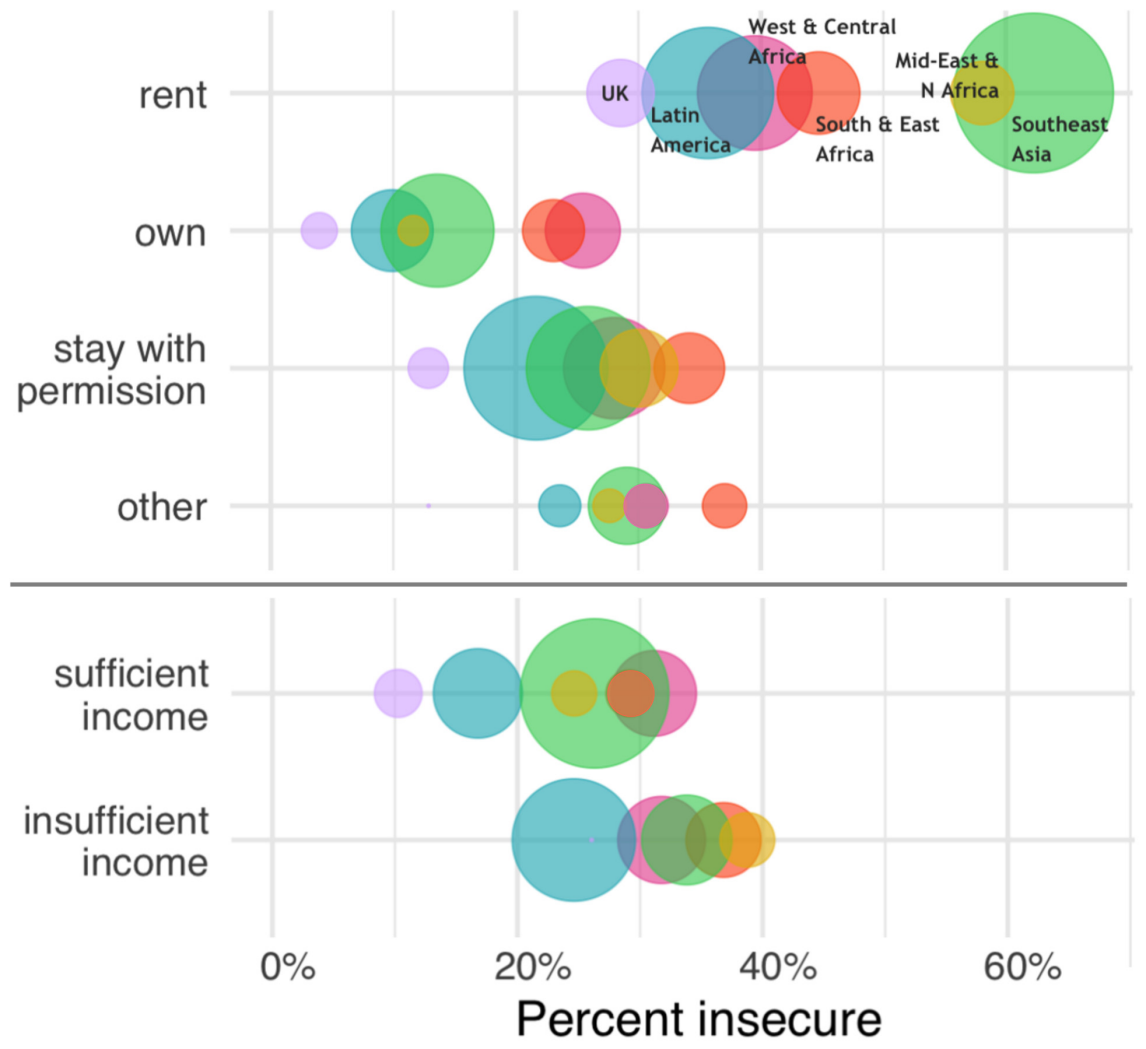
Perceived housing tenure security by tenure form (top) and income level ^a^ (bottom) for urban populations in 33 countries across 7 global regions ^b^ in 2018. Circles in each category are proportional to population size. a ‘Sufficient income’ = getting by on or living comfortably or very comfortably on current income; ‘Insufficient income’ = difficult or very difficult to live on current income. b UK: United Kingdom; Latin America: Bolivia, Colombia, Costa Rica, Ecuador, Honduras, Mexico, Peru; West and Central Africa: Benin, Burkina Faso, Cameroon, Cote d’Ivoire, Ghana, Liberia, Niger, Nigeria, Senegal; South & East Africa: Namibia, Kenya, Madagascar, Malawi, Mozambique, Rwanda, Tanzania, Uganda, Zambia; Mid-East and N Africa, Middle East and North Africa: Jordan, Morocco, Tunisia: Southeast Asia: Cambodia, Indonesia, Vietnam, Thailand. Countries in East and South Asia including China and India were included in the 2018 Prindex database which limits its global representation. *Data source: Prindex, 2018*.

In our summary of global data on perceived tenure security, property owners reported more secure tenure than other forms of tenure, regardless of region, but most notably in regions where housing markets tend to be more formalized ( Figure 2a). Households reporting insufficient incomes also reported having less secure tenure, on average, with the exception of West and Central Africa where it was similar for households with sufficient versus insufficient income ( Figure 2b). As discussed above, any form of tenure (owned, rented, or other) can span a range of secure or insecure conditions. Renters more often experience tenancy arrangements that are not protected in law or practice, allowing easier eviction or insufficient upkeep of a dwelling by landlords. For homeowners, tenure insecurity may mean a high risk of foreclosure or that their home or the land it is on is in dispute. Still, Figure 2 shows general patterns that indicate, on average, owners feel most secure, renters feel least secure, and those staying somewhere with permission or have some other arrangement fall in the middle. 

*Industrialized contexts.* Tenure insecurity can affect industrialized urban contexts in a variety of ways. Recent work shows how water, sanitation, and energy issues, often perceived as mainly problems in the Global South, are also features of tenure insecurity in North America ( Jessel *et al.,* 2019; Meehan *et al.,* 2020). Securing housing tenure for renters is of increasing importance as the private and informal rental sectors grow and rents increase ( Arundel & Doling, 2017). In the UK, for example, rents have risen twice as fast as wages over the last decade ( Bailey, 2020). Securing tenure for renters and landlord-owners alike can incentivize higher housing quality standards due to increased rental or (eventual) sale prices, in addition to legal repercussions of violating legal building codes or regulations. In the U.S., for example, urban housing policies to protect renters are well-known, but significant variation in implementation at city and state levels leaves many renters vulnerable to eviction and poor quality housing ( Moran-McCabe *et al.,* 2020). Inspections of rental properties for safety and quality are legislated in the U.S. but tend to only happen in response to a tenant filing a complaint. In the absence of legislative protections that prevent retaliatory evictions or other retaliatory actions (e.g., harassment by the landlord or increased rents), tenants are less likely to report housing problems ( Gousy, 2014). In the UK, measured housing conditions in the private rental market were worse than any other tenure type ( Crook, 2002).

Social housing units with below-market rents and strict occupancy qualifications are intended to supply low-income and working-class families with affordable, well-built, and secure housing in many countries. In the 1990s, such social renters in the UK fared better than private renters in the size of living space and multiple features of housing quality including natural light, building material quality, heating and insulation, and air quality ( Pevalin *et al.,* 2008), which all have implications for health. However, in the past several decades, urban housing shortages and privatization have reduced the supply of social housing and raised rents ( Cowan & McDermont, 2021). In 2017, the use of low-cost external cladding on London’s Grenfell Tower – a large social housing unit – reduced building expenses but its high-flammability contributed to a devastating fire that killed 72 residents. The local council argued that the same building materials were used throughout England, while residents argued that the cost-cutting measures were unnecessary and reflected a general neglect of housing conditions. Even before the fire, Grenfell residents expressed other concerns about various unsafe building conditions to the local council, but some complaints were ignored or even met with threats of legal action ( Minton, 2017). Alternative and affordable housing options were scarce, leaving residents with little autonomy to improve their living conditions. The circumstances of the Grenfell tragedy will likely remain contested, but this case highlights how lack of empowerment in tenure insecure communities contributes to disparities in housing quality and safety, even in very wealthy cities.

***Better housing and living conditions can improve health.*** The importance of housing conditions in maintaining good health is well established and lays the foundation for many ‘first principles’ of public health. Florence Nightingale warned Londoners over 150 years ago that “badly constructed houses do for the healthy what badly constructed hospitals do for the sick” ( Nightingale, 1868). Convincing empirical evidence linking housing conditions and human health has since accumulated, most of can be generalized across most settings. We thus present this evidence integrated across developed settings.

The 19 ^th^ and 20 ^th^ centuries saw dramatic reductions in infectious diseases like cholera and tuberculosis in then-emerging western nations from housing improvements, including infrastructure upgrades, better ventilation, pest control, and reduced crowding ( Krieger & Higgins, 2002; Shaw, 2004). Similar trends were recently observed between housing improvements and infectious disease, specifically malaria, in sub-Saharan Africa ( Tusting *et al.,* 2017). The use of less-polluting household energy and improved indoor warmth can improve pregnancy and birth outcomes ( Amegah *et al.,* 2014) and reduce cardio-respiratory diseases and susceptibility to infection across a variety of contexts ( Baumgartner *et al.,* 2020; Thomson *et al.,* 2013). Investments in housing materials provide security and serve as a defence against crime, and are associated with increased usable space, greater privacy, and better mental health ( Pevalin *et al.,* 2008; Thomson *et al.,* 2013). Reductions in falls and other injuries were observed after low-cost home improvements including the installation of safety features like handrails for steps and stairs and outside lighting ( Ige *et al.,* 2019; Turner *et al.,* 2011), which can substantially reduce health care costs ( Garrett *et al.,* 2014) and improve personal autonomy, particularly for older adults ( Bunn *et al.,* 2008). Importantly, the quality of neighbourhood infrastructure and services beyond an individual house also contribute to health ( Newman & Holupka, 2018), and health benefits can accumulate as more households in a neighbourhood invest in improvements like clean energy, sanitation, drainage, and pest control ( Farley *et al.,* 2006; Jung *et al.,* 2017; Shah *et al.,* 2011).

Direct evidence of housing tenure security and health is largely based on homeownership, which assumes security, or assessment of homes that gained access to registered property or land title ( Dietz & Haurin, 2003). Observational studies in Europe showed that, compared with renters, homeowners had lower rates of mortality and chronic diseases including cancer and cardiovascular diseases ( Fox & Goldblatt, 1982; Leon, 1989). Yet in many cases, tenure status was viewed as an indicator of age, social class, or wealth rather than an explanatory factor on its own ( Carr-Hill *et al.,* 1992), even though ownership-health associations were observed in studies that controlled for these socio-demographic factors ( Laaksonen *et al.,* 2008; Macintyre *et al.,* 2001; Pollack *et al.,* 2004).

Security of tenure may also confer feelings of safety, control, autonomy, and power to exercise choice, which are associated with better mental health outcomes and may be more fundamental social determinants of health ( Burgard *et al.,* 2012; Dupuis & Thorns, 1998; Marmot *et al.,* 2011; Pevalin, 2009; Whitehead *et al.,* 2016). Occupants of a resident-controlled social housing unit in London, for example, reported better housing conditions and higher life satisfaction and wellbeing compared with previous occupancy under landlords, particularly in relation to a sense of being settled and having control over destiny ( Ambrose & Stone, 2010). In suburban Buenos Aires, allocation of land titles to squatter homes improved social connections within communities and reduced anxiety about displacement compared with controls ( Van Gelder, 2009)

***Tenure secure neighbourhoods have better access to health-promoting services.*** Security of tenure can also be a catalyst in stabilizing neighbourhoods by improving access to government services and infrastructure. Formalisation of tenure through legal rights can lead to a better functioning housing market, turning housing into an asset for household wealth accumulation and investment, which can lead to increases in overall wealth and community investment ( Durand-Lasserve & Royston, 2002). In Pune, India, informal neighbourhoods allocated more secure tenure through a government formalisation program were more likely to qualify for government assistance and obtain access to urban services than communities not in the program ( Nakamura, 2017).

By comparison, neighbourhoods without secure housing tenure are more likely to be excluded from aid distribution and post-disaster recovery programs, thus exacerbating the detrimental impacts of natural disasters. In Puerto Rico, just 40% of the over 1 million claims for disaster relief following Hurricane Maria were granted by the U.S. government, with the primary reason for denial being the inability to provide proof of ownership ( Garcia, 2019). Formal property title was less common in Puerto Rico due to informal construction, lost documentation, and high upfront costs of formalisation ( Garcia, 2019). Tenure insecure renters can be particularly disadvantaged after disasters. After the 2001 earthquake in Gujarat, India, households with documented property rights could access public assistance, whereas renters faced higher rents and higher rates of eviction from reduced housing stock ( Mukherji, 2018).

Resolving property or land disputes may be necessary to reduce conflict and increase tenure security in rapidly urbanizing settings, but the process is often fraught with conflict or violence ( Estrin, 2021; García-Godos & Wiig, 2018). Two-thirds of India’s 7.5 million civil court cases in 2016 were related to property disputes and most involved poor households ( Daksh India, 2016). In Ghana, conflict and disagreements among family members was a primary reason for housing tenure insecurity among renters and owners ( Prindex, 2019), and in Accra specifically, property disagreements are a primary cause of conflict ( Ghana Statistical Service, 2014). Disputes can persist for generations and form entrenched social and political views. Cities must therefore carefully pay attention to the process by which property disputes are resolved, who benefits, and who fails to benefit from tenure formalisation.

### Cross-cutting issues in tenure security and health

***4.2.1 The urban poor are most impacted.*** Among urban households in 33 mostly low- and middle-income countries that perceived their housing tenure as insecure, nearly half (47%) identified as being poor ( Figure 2). The shortage of adequate and affordable urban housing is an underlying driver of tenure insecurity, regardless of the overall level of economic development, though contexts can differ considerably. In North America and Europe, many households faced difficulties in maintaining homeownership after the 2008 Global Financial Crisis, where nearly 8 million home foreclosures occurred over the next decade ( Hilber & Schöni, 2016) with Black and Latinx communities being disproportionately affected ( Rugh, 2015). The housing crisis, coupled with a decline in social housing and increased demand for urban mobility by younger generations, contributed to a shift toward rental housing where tenures are almost universally less secure, particularly for low-income households. Evictions are more common in racialised communities in the U.S. ( Desmond, 2012). The Bronx borough in New York City, for example, has the highest percentage of Black and Latinx households and also the highest rates of severely rent-burdened tenants and evictions ( Mironova, 2018). Women are particularly vulnerable: in Milwaukee (U.S.), low-income women were evicted at higher rates than men due to lower incomes, children, and gender dynamics with mostly male landlords ( Desmond, 2012).

Tenure challenges are exacerbated in transitioning economies where government-backed social protections are limited and planned development is sporadic. In Latin America – where the level of urbanization is already around 80% – progressive home-ownership policies for low-income families created affordable housing stock at the urban periphery in many cities, but often without accompanying infrastructure including schools, businesses, or affordable transport ( Murray & Clapham, 2015). Workers living in suburban neighbourhoods in Goiania (Brazil), Barranquilla (Colombia), and Puebla (Mexico) reported feeling more socially isolated and spending more time and money on commutes than workers of similar incomes living closer to city centres ( Libertun de Duren, 2018). Affordable housing needs to be accompanied by sufficient access to urban services for households to realize the full benefits of more secure tenure.

Informal settlements often outpace citywide growth in South Asia and sub-Saharan Africa, where over 80% of global urban growth in the next decade will occur. In the absence of legal property title or other protections, households in informal systems are easy targets for eviction and displacement since their occupancy status can simply be ignored by authorities. Provision of secure tenure is considered one of the most effective tools for alleviating poverty in informal and slum communities, making it a common precondition for slum ‘upgrading’ projects ( Durand-Lasserve & Royston, 2002). Further, securing tenure over entire informal communities was shown to bestow comparatively greater benefits on the lowest income households ( Friedman *et al.,* 1988).

Still, a critique of many slum development projects is that they typically promote ownership and ignore renting, which may be a preferred tenure option for some ( Gilbert, 2003). Rental housing is more prevalent in informal communities than is often acknowledged and is an important form of housing for the urban poor who cannot afford ownership in the city or desires greater urban mobility. In Nairobi and Dhaka, Bangladesh slums, for example, most households rent (92% and 75%, respectively) rather than own (8% and 22% respectively) ( Bangladesh Bureau of Statistics (BBS), 2014; Talukdar *et al.,* 2010). Renting often comes with greater flexibility and less liability than ownership, making it desirable for people in transition ( Alini, 2021). Ensuring safe and healthy living conditions for renters is challenging but deserves much more attention, especially in developing and transitioning economics.

***Challenges in establishing causality for secure tenure and health.*** It is challenging to establish the causal effects of tenure security on physical or mental health, particularly given that most studies on this topic - and on housing and health more generally - are observational and lack control groups or statistical adjustment for key confounding variables ( Dietz & Haurin, 2003). Observational designs have two important limitations. First, population characteristics including social class and wealth tend to vary by level of security ( Macintyre *et al.,* 1998) and can themselves increase risk of exposures to poor quality housing and environmental and social risks ( Macintyre *et al.,* 2001). Second, reverse causality is of concern in cross-sectional studies that assess tenure security at the same time as the health, i.e., poor health might affect income or employment and thus influence the ability to buy a home or pay rent. Particularly compelling, therefore, are randomized or quasi-randomised study designs that allow for the observation of health outcomes in households that gained access to more secure tenure compared with similar households that did not, with health assessments conducted before and after intervention. For example, titling programs that granted official documentation of property ownership to households in Lima, Buenos Aires, and Montevideo (Uruguay) had effects on health across multiple age groups, including lower teenage pregnancy rates, more optimal child weight, and reduced risk of cardiovascular disease and diabetes ( Galiani & Schargrodsky, 2004; Gandelman, 2010; Vogl, 2007). The program in Lima was inclusive of women and had the additional effect of reduced fertility rates, with some evidence that improved female bargaining power was a mediator ( Field, 2003). Building on this limited empirical work, future studies might evaluate community and other large-scale social programs with housing components intended to improve security (e.g., housing subsidies, purchasing rules) to assess whether households or communities exposed to these policies perceive their tenure as more secure and whether they experience the hypothesised reductions in exposure to social, environmental, and housing risks and improvements in health and wellbeing.

***Housing tenure insecurity during the COVID-19 pandemic.*** The ongoing COVID-19 pandemic further highlights how actions to improve tenure security can protect low-income and working-class residents. To slow the spread of the virus, many cities went into lockdown and closed schools and non-essential workplaces. Tens of millions of workers lost their incomes without severance or benefits and reported being at risk of missing rent or mortgage payments ( Busby, 2020). Many cities and several countries passed emergency legislation to shelter the homeless and protect homeowners from foreclosure, including suspension of mortgage payments, mortgage relief, and short-term moratoriums on foreclosures ( Collins & Betpera, 2020; Her Majesty's Treasury (HM Treasury), 2020). Other places issued safeguards to renters including rent deferrals or waivers and temporary halts on evictions ( Collins & Betpera, 2020; Her Majesty's Treasury (HM Treasury), 2020). Though, security in rental tenure is highly subject to local politics even in the pandemic. During the eviction moratorium in the US, for example, housing advocacy groups found that landlords still found loopholes to evict tenants and some judges ignored the moratorium or questioned federal jurisdiction on this issue entirely ( Cowin *et al.,* 2020).

Importantly, protecting tenure security is also a prevention strategy against the transmission of COVID-19: lockdowns, quarantines, contact tracing, and other social distancing measures will be less effective and more controversial in cities where property disputes are common or protections for renters and owners cannot accommodate the magnitude of job loss and economic hardship resulting from the pandemic ( Benfer *et al.,* 2021).

### Policies and actions to improve tenure security in cities

Now is the time to take advantage of global disruptions like COVID-19 and reorient urban planning and housing policies to promote secure tenure. As we head into the United Nation’s newly launched ‘Decade of Action’ to achieve the SDGs, cities have the opportunity to increase tenure security in the near and long-term and, by extension, improve urban health.

Actions to improve tenure security must account for the diversity of legal, cultural, socio-economic, and political systems within which property rights operate ( Payne & Durand-Lasserve, 2012). Key policy issues in most cities include managing the balance between ownership and rental markets, developing appropriate rental safeguards, and ensuring adequate investment in housing quality. In the rapidly urbanizing contexts of cities in the Global South, policymakers must additionally grapple with fundamental tenure issues in peri-urban and slum areas. Creative and practical approaches to tenure security have been applied across a range of development and political contexts and provide important lessons. We describe some of these approaches below, while acknowledging that there is no ‘silver bullet’ solution and factors like the urban context and existing housing market must all be considered.

*Formalisation of property and land rights.* Formal documentation of land and property rights are often necessary for longer-term security and are the most common policy tools for improving tenure security in cities with more formal housing markets. Though informality does not always imply tenure insecurity, *per se* ( Thomson *et al.,* 2013), disagreements between formal versus informal rights can be a significant barrier to achieving secure tenure ( Robinson *et al.,* 2018). In cities in the Global South that are undergoing rapid expansion, for example, land on the urban fringe is subject to tenure transformation ( Durand-Lasserve & Royston, 2002). Developing structured and less bureaucratic planning processes and timely allocation of peri-urban property rights, especially before power dynamics shape the outcomes of new and competing land pressures, can help to ensure the rights and compensation for current landholders (i.e., rural farmers) and lay the groundwork for functional urban housing markets ( Adam, 2014; Owusu, 2008).

Formalizing property ownership can also involve risks, especially for the urban poor. Administrative processes and home maintenance costs can be time-consuming and cost-prohibitive, and can have financial consequences that include distress sales ( Cross, 2012), mortgage delinquency, or foreclosure ( ISSER, 2019). Landlords may need to increase rents to cover administrative costs or comply with housing regulations, displacing tenants who cannot afford to pay more ( Durand-Lasserve & Royston, 2002). At the city scale, rapid changes in ownership rates can generate speculation, inflationary pressures, and economic volatility ( Durand-Lasserve & Royston, 2002).

*Community land trusts.* Community ownership through land trusts has been used for decades, mostly in North America and Europe, to secure housing tenure while avoiding some risks of titling. Retaining collective ownership and allowing members to hold long-term leases facilitates greater autonomy in management practices ( Ambrose & Stone, 2010) and protects individual households from the municipal government or land developers ( Algoed *et al.,* 2020), and from corporations that may exploit customary forms to tenure to displace people ( Veit, 2019). Trusts differ from most other affordable housing options by allowing for wealth generation and facilitating social cohesion, both of which can be health promoting ( Hindman & Pollack, 2020). Conventional homeowners in the U.S. were eight times more likely to be in the process of foreclosure than community land trust homeowners after the 2008 housing crisis ( Thaden, 2011).

Political and popular support for community land trusts is often mixed. Individual property rights are often the norm, and land developers may hesitate to heavily invest in such projects due to perception of limited demand ( Bunce, 2016). This may change as city experiences with land trusts grow, but some trusts involve complex financial and management structures to clarify the mix of communal versus individual responsibilities. This can translate into lengthy documentation processes, and may discourage property investment, especially if members have limitations on what are perceived to be typical rights (e.g., full autonomy to sell or rent their property) ( Bassett, 2005).

Despite these challenges, trusts can enable families to remain in gentrifying neighbourhoods and can be creatively combined with other development programs. In San Juan, Puerto Rico, for example, a community land trust was established alongside environmental remediation projects in informal communities that were vulnerable to flooding ( Davis & Fernández, 2020). The flood prevention upgrades will make the communities safer and more desirable, while the Trust protects the communities from displacement and increases in land value (i.e., provides collective tenure security) while also providing residents with affordable housing, home mortgages, and property inheritance ( Davis & Fernández, 2020).

*Strengthen rental rights and security*. Few governments have formulated policies to safeguard renters’ rights, reflecting general neglect to the conditions of tenants and landlords even as renters represent an increasingly large slice of urban tenures across high- and low-income countries. Thoughtful rental policies and regulations can both protect the tenure security of renters and incentivize high-quality housing. In New York City, property owners that have been charged with a retaliatory eviction (i.e., an eviction after a tenant complains about safety violations or joins a tenant rights group) are unable to make changes to tenancy agreements (e.g., increasing rent or reducing services) and cannot renew the tenancy for a year ( Lowe, 1979). Rent control policies in Switzerland ( Hilber & Schöni, 2016) prohibit landlords from increasing rents unless they can formally document that costs increase (e.g., due to renovation costs or interest rates). Renters are also given 30 days after moving into a new property to submit a claim that rent is too high. These policies can protect tenants against irregular evictions and help maintain high-quality rental housing, though they do have limitations. Renters seeking to move, for example, must engage in time-consuming and costly search efforts, which can be challenging for the poor, elderly, and tenants with disabilities.

Improving access to information on legal rights can also help to ensure that renters are claiming all they are due and that they are aware of available services. Low knowledge of tenancy rights among private renters in Accra contributed to low demand for better sanitation facilities, and landlords subsequently felt no obligation to provide or maintain them ( Awunyo-Akaba *et al.,* 2016). Legal assistance to renters is another mechanism. New York City passed a Right to Counsel law in 2018, the first of its kind in the U.S., that provides low-income tenants facing eviction with free access to a lawyer, with plans to extend the benefit to all tenants by 2022 ( Petersen, 2020). Before the law, landlords appeared with council in over 90% of eviction proceedings compared with less than 1% of tenants. After the law passed, 32% of tenants brought lawyers to their hearings ( Petersen, 2020). Other U.S. cities including San Francisco and Philadelphia have since passed legislation with similar protections for renters. Though, exposure to justice system while being processed as a plaintiff or defendant is also associated with worse mental health ( Clemente & Padilla-Racero, 2020).

*Recognition of de facto systems*. Increased recognition of *de facto* land or housing right regimes can be near-term, inexpensive mechanisms for improving tenure security in low-income and some middle-income cities with less formalized housing markets ( Porio & Crisol, 2004). In cities where public works are more formalized, for example, tenure security can be improved over time through the accretion of documents relating to municipal taxes, utility charges, or voter registration forms ( Payne & Durand-Lasserve, 2012). In Pune, India, slum residents can retain their water tax bills and demand government re-compensation if forced to relocate ( Nakamura, 2017). These systems are still vulnerable to changes in government policy but can provide some level of near-term protection. Administration of new areas can also bring challenges ( FAO, 2012), but greater support for training professionals in surveying, land administration, and the emergence of international guidelines for this type of recognition can help ease transitions ( IFC, 2012). Digitization, improved access to and transparency of records, and reductions in registration time and costs can further promote tenure acquisition through these routes. For example, the implementation of digital addressing systems in informal settlements in Kolkata (India) enabled residents to obtain government-issued identification and improved their access to urban services, utilities, and banking ( Mason, 2020). 

Over the longer-term, communities can use incremental approaches to tenure where initial steps build on existing local ( *de facto*) mechanisms and subsequent measures move toward legal recognition and formal ownership. These types of hybrid approaches recognize that communities often need time to resolve property conflicts and develop the technical and administrative capacities for formalisation which gradually improve tenure security over time ( Payne & Durand-Lasserve, 2012). In Trinidad and Tobago, for example, *de facto* rights can be temporarily recognized with a “Certificate of Comfort” which assures households of a place to live, either in their current house or with the promise of another house if it was necessary to be relocated. ( Payne & Durand-Lasserve, 2012). This keeps land ownership and administration in the hands of the State and can be an initial step towards legal recognition, but can have risks. It regularises households’ relationships with the State and taxation, which may not be affordable for poor households and can stimulate land speculation ( Fernandes, 2011). In the favelas of Rio de Janeiro, for example, formalisation through property titles led to increased utility bills and developer interest, which then increased housing prices and eventually displaced families ( Arsenault, 2016; Fernandes, 2011).

### Improving integration of data and training 

Mitigating insecure tenure and its health impacts requires multi-sectoral approaches and collaboration, which is challenging when the areas of land tenure, housing quality, environment, and health have historically been academically and professionally siloed. Greater integration across these areas can produce a more nuanced understanding of what tenure security means for different populations, and how contextual conditions may mediate how outcomes are affected by land tenure. Improvements in transparency and accountability in monitoring and documenting land rights is an immediate need in both housing and land-based contexts. Harmonizing data for monitoring tenure security and evaluating interventions ( GLTN, 2016) and developing comparable indicators for land tenure security, land administration, land conflict, and sustainable land use and management ( Omusula, 2022) are also multinational priorities. 

Data limitations are another barrier that can be improved. Efforts to collect and digitize tenure- and tenure security-related data, including measures that capture and monitor tenure status and document the use of land and housing structures, are being developed and instituted at national and international levels. For example, PRINDEX now integrates questions about perceptions of tenure security into global and nationally representative Pew surveys. Further, ongoing efforts to monitor progress toward the SDGs, and here specifically target 1.4.2, are making tenure data more readily available and transparent. More local surveys, like the Survey of the Health of Wisconsin ( SHOW, 2023), are beginning to ask questions about tenure, enabling investigation between tenure security and health. Moving forward, we encourage national censuses and surveys to incorporate housing and land questions that better capture attributes related to tenure security beyond ownership. The inclusion of tenure in the SDGs targets further highlights the importance of harmonization and data that can be used to measure changes over time and the impact of interventions. 

Finally, universities and other higher education institutions can also play a role in breaking down academic silos by, for example, offering interdisciplinary degree programs and internships that train scholars who are skilled in real-world problem solving by incorporating knowledge and experience from multiple disciplines and sectors. The SDGs may be one useful lens for considering tenure security within broader societal challenges. As outlined in our conceptual model, insecure tenure is a determinant of multiple SDGs, with implications for sustainable cities, poverty alleviation, eradication of hunger, gender equality, clean water and sanitation, clean energy, and productive land use, all of which have well-established health relevance. The SDGs provide an action-oriented framework that can be used to bridge the gaps between academic disciplines that not have historically considered tenure security and also create partnerships between governments, business sectors and educational institutions.

## Conclusion

Our conceptual model can be used by cities around the world to motivate and inform urban planning and housing policies that contribute toward achieving the 2030 SDG Agenda. Our synthesis of the historically siloed evidence provides an important summary of the different links along the accountability chain between tenure security and health. Given the foundational role of tenure security for multiple risks and multiple health outcomes, evidence is sufficient for actions that that address tenure security in municipal policy and planning. This seems especially important to increase access to healthy housing for the urban poor, and during attending crises such as the COVID-19 pandemic. There are well-established policy measures that can improve tenure security, though we acknowledge that the political economy around these alternative measures will often be hard to change and that tenure security is a systems issue that exists for the long-term. Failure by the environmental and global health communities to more widely recognize improved tenure security as a health intervention is a lost opportunity and one that, if it continues, may hinder the ability to meet the SDGs by the end of this decade.

We also identified gaps in the literature on direct evidence that policies and programs aimed at improving housing tenure security can improve population health, particularly for renters who comprise an increasingly large share of urban tenures. Moving forward, there are many areas for intersectoral collaboration between researchers and practitioners to improve and inform housing policy including the collection and collation of high quality data, improved communication between researchers, decision makers, and citizens, and more rigorous study designs –– be they randomised, quasi-randomised, or observational. These are key elements to developing a deeper evidence base for the causal links between housing tenure security and health, the factors that link them, and the potential risk or unintended consequences.

## Ethics approval

The workshops in Ghana were registered as minimal risk by the Research Ethics Office at Kings College London (MRA-18/19-9126) and approved with other project activities by the University of Ghana ethics committee (ECH149/18-19). The stakeholder workshop was exempt from ethics review at University of British Colombia based on the Tri-Council Guidelines Policy #TCPS 2 (2018).

## Data Availability

The data used in this study are feely and publicly available at https://www.prindex.net/data/.
