# Supplementary material for: Synthesizing the links between secure housing tenure and health for more equitable cities
Source: Wellcome Open Res. 2023 Mar 13;7:18. Originally published 2022 Jan 20. [Version 2] doi: 10.12688/wellcomeopenres.17244.2 (PMC10466000; doi:10.12688/wellcomeopenres.17244.2)
Supplement: Peer review history

# Reviewer report: Reviewer response for version 2

**DOI:** 10.21956/wellcomeopenres.21111.r64662

**Published:** 2023-08-29

**Authors:** Lucy Telfar Barnard

**Affiliations:** 1 Department of Public Health, University of Otago, Dunedin, Otago, New Zealand

I'm not clear on why this is an Open Letter rather than an original research paper? However, that's more an editorial decision for the publisher. 

 I like the succinct description of substance and security of tenure.

 I feel like your introduction undersells the extent and import of the work you've done - which only becomes apparent in the Methods section!

 In figure a you mention the risks of distress sales and pricing out of poor households - the risk of pricing out poor households can be averted to some degree by the provision of public housing with security of tenure. Your figure mentions "private investment in housing and services" and "public investment in neighbourhood infrastructure and urban services", but doesn't appear to (and should) include "public investment in housing and services"? The point could also be mentioned in the text covering the same issue - possibly it is not included because it wasn't raised in the focus groups? If that's so, then I would expect to see it mentioned later as a limitation.

 Linking secure tenure with improved livelihoods and living conditions. In this section, I'm wondering whether the benefits of secure tenure are in part also a product of the substance of the tenure (i.e. ownership) as well as the security of the tenure - do the same benefits accrue if the occupants have secure rental tenure, or only secure ownership? The ownership issue is raised in the second paragraph and becomes clearer as the section continues, but wants to be clarified in the first paragraph as well .

 At this point in reading it emerged that the overall structure didn't match the conceptual model. As you'd provided figure a as a conceptual model for how housing tenure security and health are linked, I would have expected to see the results section presented according to that framework. Instead, the topic headings in the results section - and their content - while interesting, don't demonstrate a clear sense of direction. I would have liked to have seen some further exploration of the evidence for the conceptual model: which parts of the model have been investigated in the evidence, and how weak or strong were the findings; and where are there still gaps or more work needed in the evidence? Gaps are identified in the conclusion, but I would like to see these placed in the context of the conceptual model, with further illustration if necessary/helpful. 

 I hope that by restructuring content to match the conceptual model, it may become clearer which parts of the remaining content are more peripheral to the main thesis. Alternatively, if the results are to be presented in a way that doesn't follow the conceptual model, then more information is needed on how results sections were selected.

 The contents of this open letter are important and far-reaching, and everything you've written is important, valuable, and well-supported by the evidence. It's only the structure that I think could be strengthened.

 Language points (apologies in advance for linguistic pedantry):

 Introduction

- I think you want "unwillingly displaced" rather than "unwilfully displaced" (also appears in Abstract). "Unwilfully" would imply that the people who displaced them didn't do it on purpose, whereas I think you mean to say that the people being displaced were unwilling to be displaced.
- The second sentence in the last paragraph is tortuous. Could it perhaps be separated or simplified? Or even some commas?
- What is meant by secure housing tenure?
- "How secure one feels in one's tenure" (rather than "their tenure"): I'm a fan of "their as a gender neutral option, but you've already gone for "one" here, so "their" isn't necessary.
- "relates _to_ various contextual and..."

Methods:

- I don't think you mean you "visualised" published data? That would imply you imagined what it might look like. I think you mean some other word here - "illustrated", perhaps? Although we perform data visualization, that doesn't mean we visualize data.

Results:

- I suggest a full stop in the first sentence - "...that impact urban health. The model is the output of..."
- (Figure 1. health is misspelt as "healtha" in the title).

Does the article adequately reference differing views and opinions?

Yes

Are all factual statements correct, and are statements and arguments made adequately supported by citations?

Yes

Is the Open Letter written in accessible language?

Yes

Where applicable, are recommendations and next steps explained clearly for others to follow?

Partly

Is the rationale for the Open Letter provided in sufficient detail?

Partly

Reviewer Expertise:

Rental housing regulation and policy, particularly rental housing quality standards; housing and health, particularly infectious and winter illnesses. Some work on demography of eviction.

I confirm that I have read this submission and believe that I have an appropriate level of expertise to confirm that it is of an acceptable scientific standard, however I have significant reservations, as outlined above.

# Reviewer report: Reviewer response for version 1

**DOI:** 10.21956/wellcomeopenres.19060.r51128

**Published:** 2022-07-06

**Authors:** Rebecca Bentley

**Affiliations:** 1 Centre for Health Policy, Melbourne School of Population and Global Health, University of Melbourne, Melbourne, Vic, Australia

The benefits of conceptualising housing as a system in the way that we treat it as a social determinant of health is a step forward in healthy housing research. This paper draws on stakeholder interviews and scoping of the literature (reported as a narrative) to propose a conceptual framework that can be used to guide action towards an equity-informed urban health agenda internationally. It is a timely and necessary contribution to the research literature.

 This is an excellent paper. My comments to the authors are focused on clarifying the conceptual diagram:

1. The conceptual diagram currently does not incorporate intersecting aspects of social positions and identities that are impacted by housing insecurity and that contribute to how health inequalities are shaped through our housing system (e.g., disability, gender, refugee/migrant status, first nations identify, precarious employment).
2. The conceptual framework is generalised across life stages. Given that security issues and their benefits for health change as people age (and their health changes) and that the consequences of secure tenure change with age (and in relation to characteristics of social welfare systems) it is important to signify this dynamic element in the conceptual diagram. The current format is static in this respect.
3. Systems level interventions to improve health are not easy to identify in the model in its current format. The model does a good job of laying out the findings from focus groups but doesn’t take the next step of summarising key aspects of tenure systems that should be reviewed with a health and health equity lens. For example, ‘better housing structures and building materials’ is descriptive. This housing system aspiration could be achieved through tightening building codes, having minimum standards in the rental sector, etc. I suggest simplifying and universalising some of the text in the conceptual diagram. In this instance, an alternative label that also gives direction might be ‘Use of safe housing structures and building materials across the sector’, and suggested mechanisms could be through setting standards and regulations. This type of label will also map more directly onto any matching data surveillance that is being established on tenancy security internationally.
4. Interactions of the housing security system with other important systems (including the justice and social welfare systems) are currently absent from the model but, I think, are necessary for planning jurisdiction specific responses.
5. I find the exposures and improvements section of the framework confusing. It would be useful if there were additional text in that more clearly describes the distinctions between these categories and how they interact/overlap and gives direction on how to use them when applying the conceptual framework.

Does the article adequately reference differing views and opinions?

Yes

Are all factual statements correct, and are statements and arguments made adequately supported by citations?

Yes

Is the Open Letter written in accessible language?

Yes

Where applicable, are recommendations and next steps explained clearly for others to follow?

Yes

Is the rationale for the Open Letter provided in sufficient detail?

Yes

Reviewer Expertise:

Social epidemiology, housing and health

I confirm that I have read this submission and believe that I have an appropriate level of expertise to confirm that it is of an acceptable scientific standard, however I have significant reservations, as outlined above.

# Response

**Published:** 2023-02-14

**Authors:** Jill Baumgartner

**Affiliations:** McGill University, Canada

**The conceptual diagram currently does not incorporate intersecting aspects of social positions and identities that are impacted by housing insecurity and that contribute to how health inequalities are shaped through our housing system (e.g., disability, gender, refugee/migrant status, first nations identify, precarious employment).** To address this comment, we edited to the conceptual diagram to include individual, social, and infrastructure buffers that can impact vulnerability and that may increase or decrease the health benefits of more secure tenure for certain population groups.   We also added the following text and reference to the manuscript which highlight the points about vulnerability made by the reviewer: *However, the benefits of secure tenure may not be the same for all population groups where individual (e.g., age, gender, disability, race/ethnicity, indigeneity), social (e.g., racism and other oppressive structures, poverty), and infrastructure (e.g., health care) factors can influence vulnerability independently or jointly and thus affect the potential health benefits gained from more secure tenure, as has been demonstrated in studies of ecosystem change and health (Myers & Patz, 2009).* Reference added: Myers, SS and Patz, JA: Emerging threats to human health from global environmental change. Annual Review of Environment and Resources. 2009; 34(1), pp.223-252.

**The conceptual framework is generalised across life stages. Given that security issues and their benefits for health change as people age (and their health changes) and that the consequences of secure tenure change with age (and in relation to characteristics of social welfare systems) it is important to signify this dynamic element in the conceptual diagram. The current format is static in this respect.** To address this comment, we added age as one of the buffers around health in the framework and included this in the text (please refer to response above).

**Systems level interventions to improve health are not easy to identify in the model in its current format. The model does a good job of laying out the findings from focus groups but doesn’t take the next step of summarising key aspects of tenure systems that should be reviewed with a health and health equity lens. For example, ‘better housing structures and building materials’ is descriptive. This housing system aspiration could be achieved through tightening building codes, having minimum standards in the rental sector, etc. I suggest simplifying and universalising some of the text in the conceptual diagram. In this instance, an alternative label that also gives direction might be ‘Use of safe housing structures and building materials across the sector’, and suggested mechanisms could be through setting standards and regulations. This type of label will also map more directly onto any matching data surveillance that is being established on tenancy security internationally.** As suggested, we edited the model and tried to combine bullet points (when feasible) and simplify the text, including the suggestions by the reviewer about safe housing and building materials. Please see edits to Figure 1 Generally we try to strike a balance between providing generalized insights into the mechanisms linking tenure security and health without being overly prescriptive about the specific policy solutions or interventions, since these are likely to vary across and even within cities and regions. Thus, we discuss interventions like minimal rental standards in the Discussion, but did not include in the model because it might be a solution for one community but not others (e.g., rural areas where rentals are less common). We edited a sentence to address this: *The conceptual model provides generalized insights into the mechanisms linking tenure security and health without being overly prescriptive about the specific policy solutions or interventions, since these are likely to vary across and even within cities and regions. We intend for it to be general enough to apply broadly across contexts while acknowledging that the relative importance of different pathways is likely to differ greatly by place or population.*

**Interactions** **of the housing security system with other important systems (including the justice and social welfare systems) are currently absent from the model but, I think, are necessary for planning jurisdiction specific responses.** To address this comment, we added access to social services and a just legal system to the factors that influence vulnerability in the framework. We also discuss the importance of the justice and social welfare systems for tenure security in the manuscript (see sections on ‘Strengthen rental rights and security’).

**I find the exposures and improvements section of the framework confusing. It would be useful if there were additional text in that more clearly describes the distinctions between these categories and how they interact/overlap and gives direction on how to use them when applying the conceptual framework.** To address this comment and better differentiate between improvements/upgrades and exposures, we added the following text: *To ease the interpretability of our conceptual model, improvements and exposures were separated and categorized, where improvements describe the physical, economic, and social changes or upgrades to homes and neighborhoods and exposures describe the downstream risk factors for health that can be affected by these changes.* We also made major edits to the earlier section describing the conceptual model: *Our model describes the benefits of households and communities having more secure tenure secure which includes greater autonomy, feelings of safety and control, and better access to credit and other financial resources. Tenure security can empower households and communities to demand better infrastructure and urban services and to engage in local governance. These factors can facilitate improvements to home and neighbourhood safety and quality of infrastructure and increase access to urban services, which can reduce exposures to environmental, social, and housing risks (e.g., flooding, pests, pollution, crime and violence, unsafe roads and play areas) that have well-established impacts on health.*

# Reviewer report: Reviewer response for version 1

**DOI:** 10.21956/wellcomeopenres.19060.r48226

**Published:** 2022-04-22

**Authors:** Kalpana Balakrishnan

**Affiliations:** 1 Department of Environmental Health Engineering, Sri Ramachandra Institute of Higher Education and Research, Chennai, Tamil Nadu, India

The authors have chosen a very pertinent and yet a little addressed topic that is critical for creating healthy communities and cities. The home micro-environment accounts for greater than 40% of time spent by individuals, over the course of their life-time and yet remains the least studied with respect to environment and health. Investments by the individual/state towards improving the home environment may be influenced considerably by evidence of linkages between the quality of home environment and individual/population health. But the willingness to make these investments is influenced by the actual and/or perceived sense of security/control over the tenement. Tenure security thus is a powerful concept that integrates the political, economic, cultural, social and legal macro-environments with individual/societal behavior. The authors do a brilliant job of justifying this rationale for why this is important. They substantiate whatever has been described adequately. In particular, the sections on linking better housing and living conditions and health; tenure security and health and the challenges in establishing causality are presented in the best possible light. The language and writing is lucid and clearly articulated for a wide base of readers.

  I have a few minor suggestions that may be helpful for the authors to consider to implications of this conceptual framework for action on the ground.

1. It may be helpful to clearly articulate in the main text (results) that the conceptual model (Figure 1) came from the extensive co-production exercises and comprehensive narrative reviews undertaken by the authors. In its current position in the text, it also seems like a method used to conduct the reviews and the stakeholder discussions.
2. It may be useful to note that the Prindex Initiative results presented here does not include South Asia. Prindex and NCAER have recently launched this in India in 2021. Since this region accounts for a considerable part of the global population, Figure 2a may not adequately reflect a truly global perspective on perceived tenure security.
3. The authors note that the topics of tenure security, housing, health and environment have remained academically and professionally siloed. Survey instruments and policies concerning these are also similarly siloed. Authors can reflect on what can be done to achieve varying levels of integration between these survey tools and policies within and across countries. It may also be important for curricula in graduate schools to formally build skills in integrating the many requirements of SDGs and not focus on individual goals relevant to a program. This may break the academic silos and enable more topic based research which is definitely needed to inform regional actions.
4. Finally, it may be important to analyze very large scale Government initiated social programs concerning subsidized housing to see if elements of tenure security are adequately reflected or if it is included in IEC regarding the programs. Such programs have a long history of roll out in multiple countries in the Global South. Future research could also explicitly focus on such programmatic efforts where it may be feasible to both improve perceptions regarding tenure security and housing quality to influence environment and health.

The paper lays out a very comprehensive conceptual framework and will benefit many groups of researchers to adopt and evaluate its applicability in local and regional contexts. 

Does the article adequately reference differing views and opinions?

Partly

Are all factual statements correct, and are statements and arguments made adequately supported by citations?

Yes

Is the Open Letter written in accessible language?

Yes

Where applicable, are recommendations and next steps explained clearly for others to follow?

Partly

Is the rationale for the Open Letter provided in sufficient detail?

Yes

Reviewer Expertise:

NA

I confirm that I have read this submission and believe that I have an appropriate level of expertise to confirm that it is of an acceptable scientific standard.

# Response

**Published:** 2023-02-14

**Authors:** Jill Baumgartner

**Affiliations:** McGill University, Canada

**It may be helpful to clearly articulate in the main text (results) that the conceptual model (Figure 1) came from the extensive co-production exercises and comprehensive narrative reviews undertaken by the authors. In its current position in the text, it also seems like a method used to conduct the reviews and the stakeholder discussions.** Added as recommended. The first sentence of the Results now reads: *Figure 1 presents a conceptual model of a hypothesized chain of events through which housing tenure security can affect home and neighbourhood factors that impact urban health, which is the output of our extensive co-production exercises and comprehensive narrative review.*

**It may be useful to note that the Prindex Initiative results presented here does not include South Asia. Prindex and NCAER have recently launched this in India in 2021. Since this region accounts for a considerable part of the global population, Figure 2a may not adequately reflect a truly global perspective on perceived tenure security.** As recommended, we added the following sentence to Figure 2: *Countries in East and South Asia including India and China were not included in the 2018 Prindex database which limits its global representation.*

**The authors note that the topics of tenure security, housing, health and environment have remained academically and professionally siloed. Survey instruments and policies concerning these are also similarly siloed. Authors can reflect on what can be done to achieve varying levels of integration between these survey tools and policies within and across countries. ** To address this comment and the one below, we added a new section to the manuscript which is titled “Improving integration of data and training” which includes the following text about survey instruments and policies. *Mitigating insecure tenure and its health impacts requires multi-sectoral approaches and collaboration, which is challenging when the areas of land tenure, housing quality, environment, and health have historically been academically and professionally siloed. Greater integration across these areas can produce a more nuanced understanding of what tenure security means for different populations, and how contextual conditions may mediate how outcomes are affected by land tenure. Improvements in transparency and accountability in monitoring and documenting land rights is an immediate need in both housing and land-based contexts. Harmonizing data for monitoring tenure security and evaluating interventions (GLTN, 2016) and developing comparable indicators for land tenure security, land administration, land conflict, and sustainable land use and management (Omusula, 2022) are also multinational priorities.* *Data limitations are another barrier that can be improved. Efforts to collect and digitize tenure- and tenure security-related data, including measures that capture and monitor tenure status and document the use of land and housing structures, are being developed and instituted at national and international levels. For example, PRINDEX now integrates questions about perceptions of tenure security into global and nationally representative Pew surveys. Further, ongoing efforts to monitor progress toward the SDGs, and here specifically target 1.4.2, are making tenure data more readily available and transparent. More local surveys, like the Survey of the Health of Wisconsin (SHOW, 2023), are beginning to ask questions about tenure, enabling investigation between tenure security and health. Moving forward, we encourage national censuses and surveys to incorporate housing and land questions that better capture attributes related to tenure security beyond ownership. The inclusion of tenure in the SDGs targets further highlights the importance of harmonization and data that can be used to measure changes over time and the impact of interventions.*

**It may also be important for curricula in graduate schools to formally build skills in integrating the many requirements of SDGs and not focus on individual goals relevant to a program. This may break the academic silos and enable more topic based research which is definitely needed to inform regional actions.** Given the importance of this topic for this particular article, we added a paragraph on the integration of the SDGs into higher education, and how this approach might be useful for addressing this range of broader societal challenges: *Finally, universities and other higher education institutions can also play a role in breaking down these traditional academic silos by, for example, offering interdisciplinary coursework, degree programs, and internship opportunities that train scholars who are skilled in real-world problem solving by integrating knowledge and experience from multiple disciplines and sectors. The SDGs may be a useful lens for considering tenure security within broader societal challenges. As outlined in our conceptual model, insecure tenure is a determinant of multiple SDGs, including sustainable cities, poverty alleviation, eradication of hunger, gender equality, clean water and sanitation, clean energy, and productive land use, all of which have well-established health relevance. The SDGs provide an action-oriented framework that can be used to bridge the gaps between academic disciplines that not have historically considered tenure security and also create partnerships between governments, business sectors and educational institutions.*

**Finally, it may be important to analyze very large scale government initiated social programs concerning subsidized housing to see if elements of tenure security are adequately reflected or if it is included in IEC regarding the programs. Such programs have a long history of roll out in multiple countries in the Global South. Future research could also explicitly focus on such programmatic efforts where it may be feasible to both improve perceptions regarding tenure security and housing quality to influence environment and health.** To address this comment, we added the following text to the manuscript: *Building on this limited empirical work, future studies might evaluate community and other large-scale social programs with housing components intended to improve security (e.g., housing subsidies, purchasing rules) to assess whether households or communities exposed to these policies perceive their tenure as more secure and whether they experience the hypothesised reductions in exposure to social, environmental, and housing risks and improvements in health and wellbeing.*
